# Structural Dynamics and Molecular Evolution of the SARS-CoV-2 Spike Protein

**DOI:** 10.1128/mbio.02030-21

**Published:** 2022-03-08

**Authors:** Kyle A. Wolf, Jason C. Kwan, Jeremy P. Kamil

**Affiliations:** a Department of Pharmaceutical Sciences, University of Wisconsin-Madison, Madison, Wisconsin, USA; b Interdiscipinary Ph.D. Program in Structural and Computational Biology and Quantitative Biosciences, University of Wisconsin-Madison, Madison, Wisconsin, USA; c Department of Microbiology and Immunology, Louisiana State University Health Shreveport, Shreveport, Louisiana, USA; d Center for Excellence in Emerging Viral Threats, Louisiana State University Health Shreveport, Shreveport, Louisiana, USA; Albert Einstein College of Medicine

**Keywords:** SARS-CoV-2, adaptive mutations, coronavirus, COVID-19, evolution, genomics, glycoproteins, infectious disease, respiratory viruses, sarbecovirus, spike

## Abstract

The ongoing coronavirus disease 2019 (COVID-19) pandemic demonstrates the threat posed by novel coronaviruses to human health. Coronaviruses share a highly conserved cell entry mechanism mediated by the spike protein, the sole product of the *S* gene. The structural dynamics by which the spike protein orchestrates infection illuminate how antibodies neutralize virions and how *S* mutations contribute to viral fitness. Here, we review the process by which spike engages its proteinaceous receptor, angiotensin converting enzyme 2 (ACE2), and how host proteases prime and subsequently enable efficient membrane fusion between virions and target cells. We highlight mutations common among severe acute respiratory syndrome coronavirus 2 (SARS-CoV-2) variants of concern and discuss implications for cell entry. Ultimately, we provide a model by which sarbecoviruses are activated for fusion competency and offer a framework for understanding the interplay between humoral immunity and the molecular evolution of the SARS-CoV-2 Spike. In particular, we emphasize the relevance of the Canyon Hypothesis (M. G. Rossmann, J Biol Chem 264:14587–14590, 1989) for understanding evolutionary trajectories of viral entry proteins during sustained intraspecies transmission of a novel viral pathogen.

## INTRODUCTION

The ongoing coronavirus disease 2019 (COVID-19) pandemic is caused by the severe acute respiratory syndrome coronavirus 2 (SARS-CoV-2), a novel betacoronavirus in the subgenus *Sarbecovirus*. Since its emergence in late 2019 in Wuhan, China, the virus has caused at least 361 million confirmed infections and 5.62 million recorded deaths (1), underscoring the threat posed by coronaviruses to human and animal health (2, 3). Despite the four human coronaviruses, HKU1, 229E, NL63, and OC43, being traditionally associated with mild “common cold” illnesses (4), the 21st century has revealed the considerable threat posed by preemergent coronaviruses that circulate in various bat species.

In 2002, a sarbecovirus that ordinarily circulates in horseshoe bats (genus *Rhinolophus*) spilled into humans from masked palm civets (*Paguma larvata*) that are commonly sold in animal markets in Guangdong, China (5), causing an epidemic of viral pneumonia. By the time the epidemic was contained in 2003, the virus, now known as the original severe acute respiratory syndrome coronavirus (SARS-CoV; here SARS-CoV-1), had spread to 26 countries on five continents, infecting over 8,000 people and causing 774 deaths (6, 7). The case fatality rate of nearly 10% and apparent pandemic potential of SARS-CoV-1 provided an impetus to investigate wildlife for preemergent SARS-like coronaviruses and to revisit the pathogenic potential of the four human coronaviruses (hCoVs), which previously were assumed to cause only mild respiratory disease. It is now appreciated that seasonal hCoVs, such as OC43 and NL63, routinely cause outbreaks in long-term care facilities with high attack and mortality rates in the elderly and immunocompromised (8, 9), and a wide range of hCoVs have been identified as the causative agent in children hospitalized with pneumonia (10). Moreover, another betacoronavirus, the Middle East respiratory syndrome coronavirus (MERS-CoV), has spilled over into humans several times from camels. Since its identification in 2012, MERS has infected more than 2,500 people and caused at least 886 deaths, a case fatality rate of roughly 34% (11, 12).

Although the threat of coronaviruses to human health was not widely appreciated prior to the 2002–2003 SARS (SARS-CoV-1) epidemic, the threat these viruses pose to livestock has long been apparent. For example, the porcine epidemic diarrhea virus (PEDV) was first identified in Europe in the 1970s and has long been endemic to China (13, 14). However, in 2013, PEDV swept through the United States, causing a fatality rate of nearly 100% in piglets and nearly decimating the domestic pig population (15). The PEDV epizootic spurred development of an alphavirus RNA particle vaccine against porcine epidemic diarrhea virus (16, 17), which has been viewed as a precursor of contemporary mRNA vaccines (18). In 2016, the novel swine acute diarrhea syndrome coronavirus (SADS-CoV) killed ∼24,000 piglets across four farms in Guangdong province, China (19). Feline coronavirus (FCoV) is capable of causing disease in wild and domestic cats (20). Due to mutation and their ease of recombination (14, 21–24), coronaviruses are efficient explorers of host species and cell tropism. Therefore, coronaviruses are enduring and pervasive threats. Understanding their virology and evolution is essential to mitigating the ongoing SARS-CoV-2 pandemic as well as for establishing a toolkit to more effectively respond to future coronavirus epidemics.

## CORONAVIRUS ENTRY

The mechanistic details of coronavirus entry have been covered elsewhere (3, 25–27). Our focus here will be on how the prefusion conformation of S engages with host cell receptors and undergoes proteolytic processing to reach a fusion-competent state. Like all class I viral membrane fusion proteins, S polypeptides assemble into homotrimers, with each subunit made up of two domains. The first subunit (S1) binds host receptors (e.g., ACE2 for SARS-CoV-1, SARS-CoV-2, and NL63) and in essence serves as a chaperone for the S2 subunit, which contains the spring-loaded machinery that executes membrane fusion. In the absence of S1, S2 rapidly and irreversibly transitions to its postfusion conformation. Many, but not all, coronaviruses encode a protease cleavage site at the S1/S2 boundary; this position, fittingly, is often referred to as S1/S2 (28). Cleavage at S1/S2 increases spike flexibility and has been suggested to promote a rapid mode of entry where fusion occurs at the cell surface (29). In the case of SARS-CoV-2 S, the S1/S2 motif enables preprocessing by the proprotein convertase furin (28, 30, 31). This feature, also known as the furin cleavage site (amino acids [aa] 681 to 685 in SARS-CoV-2 S), has been suggested to be crucial to the pandemic spread of SARS-CoV-2 (32) and has been an important locus of adaptive evolution in the SARS-CoV-2 *S* gene (33–35). Additionally, all coronaviruses have a second cleavage site within the S2 subunit called S2′ (28). Cleavage of this second site is strictly required for cell entry as it exposes the hydrophobic fusion peptide that anchors in the host cell membrane during the fusion process (36–38).

**FIG 1:**
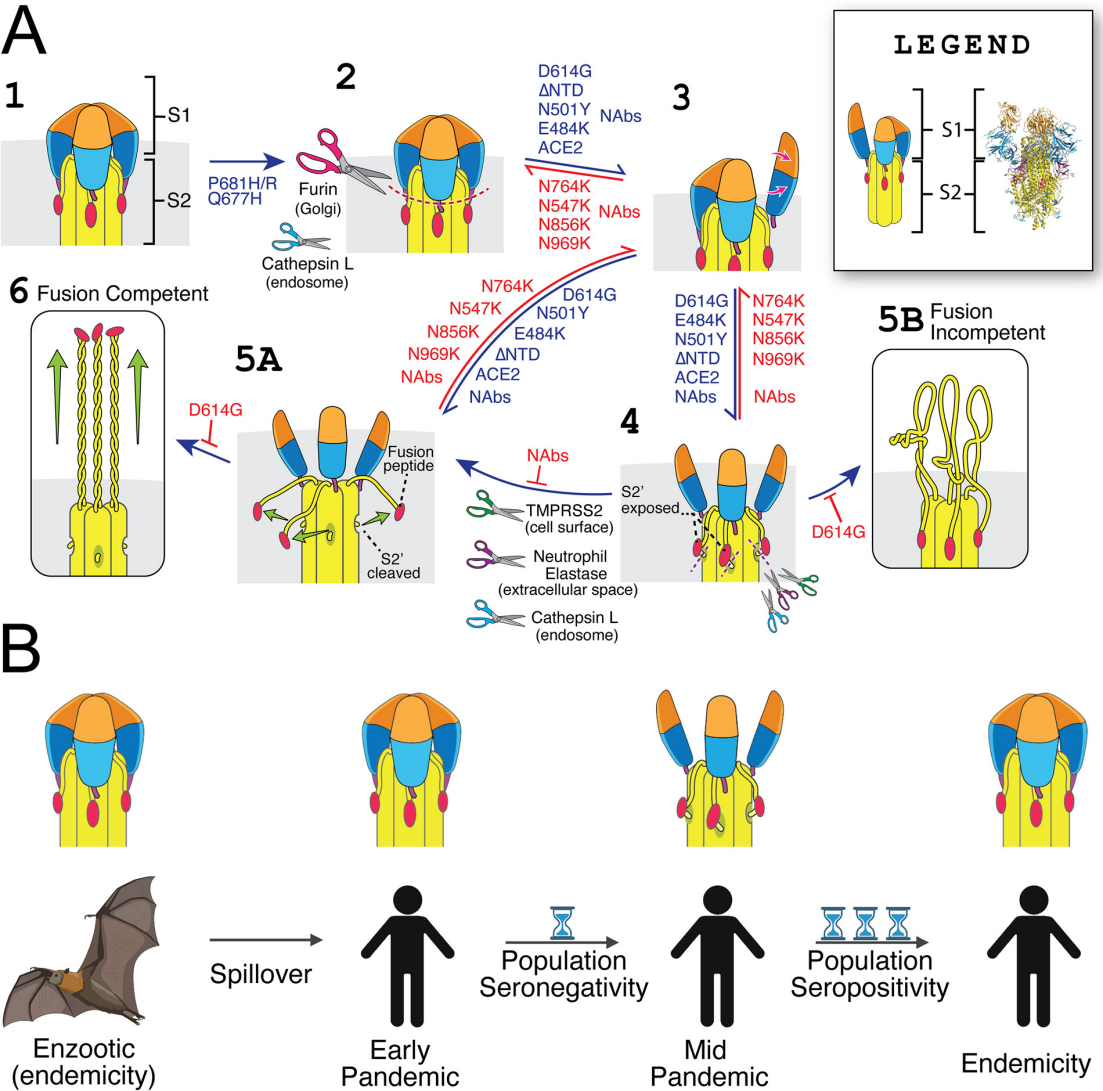
Structural and evolutionary dynamics of the SARS-CoV-2 Spike protein. (A) Model for how sarbecovirus Spike proteins achieve fusion competency and effects of common mutations. (Stage 1) An uncleaved S protein trimer. (Stage 2) Cleavage at the S1/S2 site can occur within the Golgi apparatus of an infected cell during the production of viral progeny but strictly speaking is not required for infectivity. This proteolytic processing at S1/S2 is made more efficient by a substitution such as P681H/R or Q677H. Alternatively, processing at S1/S2 could occur subsequent to egress of viral progeny, e.g., during endosomal entry. S1/S2 cleavage destabilizes the prefusion conformer, which promotes opening of RBD and the transition to stages 3 to 6. The D614G substitution, NTD loop deletions, and RBD mutations such as N501Y and E484K likewise increase RBD opening, which promotes binding to ACE2. When the three subunits of an S homotrimer simultaneously adopt the RBD open conformation, a state that is stabilized by receptor (ACE2) binding, the S2 subunit adopts increased flexibility, exposing the S2′ site for cleavage by host proteases (e.g., TMPRSS2, neutrophil elastase, and cathepsin L) (4). However, should S1 dissociate prior to S2′ cleavage (stage 5B), the S2 subunit prematurely transitions to its postfusion conformation, which is irreversible and tantamount to a noninfectious dead end. Alternatively, when S2′ cleavage occurs prior to dissociation of the S1 subunit (stage 5A), S is fully activated and competent to mediate fusion (stage 6). Therefore, stage 4 likely represents an unstable, transient state wherein S protomers can achieve fusion competency. Importantly, stage 6 must occur in close proximity to a target membrane (e.g., a host cell phospholipid bilayer) in order to achieve fusion. By decreasing the rate of S1 dissociation, the D614G substitution limits the occurrence of misfiring events (stage 5B), making fusion more efficient and offsetting stability costs of mutations that increase preprocessing at S1/S2 and/or enhance sampling of RBD open states. Neutralizing antibodies (NAbs) can impact viral entry in many different ways, depending on where they bind and how they affect S protein structure. The legend inlay indicates side-by-side PyMol rendering of a cryo-EM structure of S (PDB entry 6VYB) (30) and its cartoon interpretation; coloring is harmonized across domains. (B) The Canyon Hypothesis applied to zoonotic spillover. During circulation in populations with high rates of humoral immunity, viral entry proteins favor predominantly closed RBD configurations (112). Immediately after spillover into a population that lacks immunity, the newly emergent virus remains closely related to its ancestor and, hence, favors closed RBD configurations. During sustained transmission between seronegative individuals, large viral population sizes and wide transmission bottlenecks facilitate rapid emergence of variants that favor open RBD configurations to spread rapidly between hosts. Over time, the evolutionary entanglement between viral entry proteins and humoral immunity gradually leads to a return to closed RBDs as repeat exposures facilitate the affinity maturation of expansive antibody repertoires that are disproportionately costly to open RBD configurations. Panel B was generated using biorender.com.

In coronaviruses such as SARS-CoV-2 that contain furin cleavage sites at the S1/S2 boundary, processing at this site is thought to occur primarily within the Golgi apparatus during maturation of newly synthesized S trimers. Such preprocessing at S1/S2 is thought to prime S for efficient membrane fusion at target cells, likely by promoting open conformations that facilitate interactions with receptors and enhancing cleavage at S2′ by cell surface proteases such as TMPRSS2 (30, 39). Without an S1/S2 furin cleavage motif, S1/S2 and S2′ sites both must be processed during target cell entry (Fig. 1A). This can be inefficient and restricts the virus to the slower endocytic entry route, in which the virus relies on pH-dependent cathepsins and may be more susceptible to restriction by certain innate immune factors, e.g., interferon-stimulated genes (40, 41) such as IFITMs, which rigidify cell membranes to prevent fusion (42, 43). Proprotein convertase (furin) preprocessing at the S1/S2 site leads to a more labile and fusogenic, but less stable, spike protein (32). The receptor binding domain (RBD) more readily binds ACE2 and the S2′ site can be efficiently cleaved by surface proteases like TMPRSS2 (44), facilitating direct entry and fast growth kinetics in respiratory epithelial cells (26, 28).

## STRUCTURAL DETERMINANTS OF VIRAL ENTRY

Due to the unidirectional conformational transition characteristic of class I viral fusion proteins, they have evolved sophisticated coincidence detection mechanisms to prevent misfiring. Influenza hemagglutinin, for example, combines proteolytic cleavage with endosomal pH reductions to destabilize the interface between its receptor engaging chaperone and fusogenic subunits (45). In contrast, SARS-CoV-2, and perhaps all coronavirus S proteins, may rely on 3 RBD simultaneously adopting the open or up conformation (3 RBD open or 3 RBD up). Cleavage at S1/S2, at least in some lineages, facilitates transition to the 3 RBD up state and may further destabilize the interface between the S1 (chaperone) and S2 (fusogen) subunits (e.g., Fig. 1A, stage 4). However, certain alphacoronaviruses lack an S1/S2 site entirely (28), and the SARS-CoV-2 spike protein can transition into the postfusion conformation in the complete absence of ACE2 and without proteolytic cleavage of either its S1/S2 or S2′ site (46). Despite this, S2′ cleavage usually occurs after S1/S2 cleavage and receptor binding (47).

Structural, computational, and biochemical evidence help clarify these seemingly conflicting observations. First, the 3 RBD open state was observed only with the inclusion of the D614G mutation (48), which simultaneously promoted opening while stabilizing the prefusion conformation (49). Molecular docking simulations between TMPRSS2 and a prefusion stabilized spike protein, in which only one of the 3 RBDs is oriented upward (e.g., Fig. 1A, stage 3), can align the S2′ loop with the protease active site (50), but R815 of the S2′ loop is not accessible because in this structure it is engaged in interactions with D820 and F823 (30). However, binding to ACE2 stabilizes Spike in the 3 RBD open conformation, causing R815 to become much more exposed (49) and therefore accessible to proteases. Moreover, D614G synergizes with N-terminal domain (NTD) loop deletions to enable ACE2-independent S2′ cleavage (51). Therefore, S1/S2 cleavage, RBD opening, S2′ processing, and the postfusion conformational transition are not steps that must occur in fixed linear order. Rather, these events are allosterically regulated through the stochastic mechanism of spike stability. The 3 RBD open conformation likely represents a transient state in which the S2′ loop is sufficiently disordered to be cleaved by host proteases. Independent of S2′ cleavage, this transient state is exited when the S1 subunit dissociates and the spike undergoes an irreversible transition to the postfusion conformer or when one or more RBDs fall back down into the closed conformation (Fig. 1A, stages 5A and 6). While the transition to the postfusion conformation can occur without S2′ cleavage (46), proteolytic processing is necessary for a fusion-competent conformational transition. Receptor binding appears to stabilize RBDs in the open conformation, promoting S2′ cleavage and the transition to the postfusion state independently.

Notably, TMPRSS2 is not the only protease capable of releasing the SARS-CoV-2 S fusion peptide. For instance, neutrophil elastase (NE) is also capable of cleaving the S2′ site (52), and an elevated neutrophil/lymphocyte ratio (NLR) during early illness clinically correlates with development of severe disease (53). Although elastase release might only modestly increase cell entry in the upper respiratory tract, due to its high TMPRSS2 expression (54), it may more markedly enhance infection of cells that express ACE2 but not TMPRSS2 (55). Therefore, elevated NE levels may be a common feature of severe COVID-19 due to an imbalanced immune response that enhances S2′ cleavage within extracellular spaces. This phenomenon might promote intrahost spread, particularly in tissues with low TMPRSS2 expression. Moreover, neuropilin-1 (56) and SR-B1 (57) improve the efficiency of ACE2-dependent entry, while soluble ACE2 enables endosomal entry in ACE2 and TMPRSS2-deficient cells through the renin-angiotensin system (58). One might even speculate that extracellular NE and soluble ACE2 could facilitate surface entry into ACE2- and TMPRSS2-deficient cells.

Thus, it would appear that the pre-Omicron variants selected during the COVID-19 pandemic combine *S* mutations that increase RBD opening and overall lability with stabilizing mutations that decrease S1 dissociation. This increases sampling of and tolerance for the 3 RBD open conformation in which the S2′ site can be cleaved, which is necessary to achieve a fusion-competent state before the S1 subunit dissociates and the S2 subunit is committed to its irreversible conformational transition. This facilitates early entry and rapid growth kinetics, promoting both intrahost replication and interhost transmission.

## HOW D614G ENABLED FUSOGENICITY-ENHANCING SPIKE MUTATIONS

**FIG 2:**
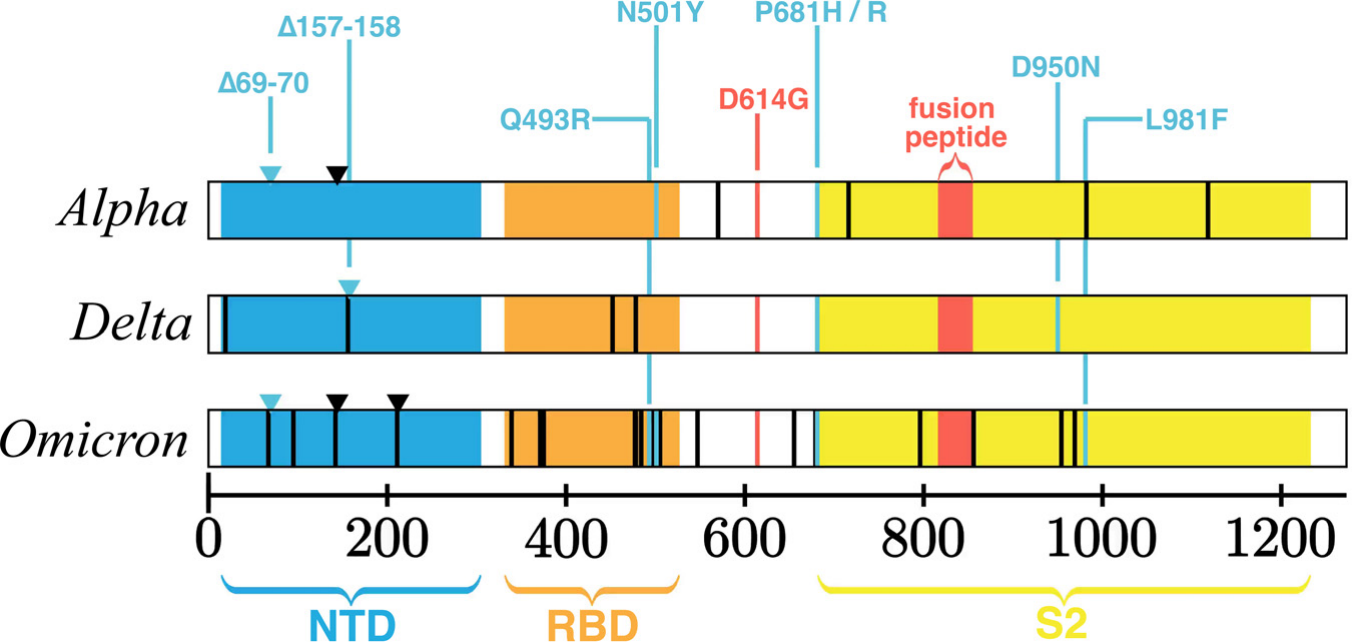
SARS-CoV-2 S domain structure and characteristic mutations of variants of concern. The SARS-CoV-2 spike polypeptide labeled by its domains and annotated for amino acid substitutions and deletions with 70% or higher prevalence in GISAID data for three prominent variants of concern, Alpha (B.1.1.7 + Q.x), Delta (B.1.617.2 + AY.x), and Omicron (B.1.1.529 + BA.x), as tabulated by outbreak.info using 12 January 2022 data.

During the first 2 years of the pandemic, we have seen the steady emergence of highly fit SARS-CoV-2 variants. Each of these variants contains a constellation of mutations, and their ultimate phenotype is determined by the epistatic interactions between these mutations. Over the pandemic, the evolution of the spike protein has undergone a series of selective phases (59). While the ancestral SARS-CoV-2 virus likely had an alanine at the 372 position of its spike protein, during the early stages of the pandemic (likely before its detection and widespread sequencing) the virus acquired the A372T mutation (60). This mutation was an early adaptation to humans that increased ACE2 affinity (60). Once the virus was circulating and differentiating into clades, the virus underwent a second selective sweep. First lineage B and then lineage A acquired the D614G mutation. This mutation did not have immediately apparent phenotypic effects beyond modestly improved ACE2 affinity, greater thermal stability, and only slightly improved transmissibility (49, 61–66). Afterwards, a host of increasingly fit variants rapidly emerged and displaced each other (67–70) (Fig. 2) (reviewed in reference 71). As each variant’s overall fitness is a complex function of many variables, we will minimize discussion of particular lineages and instead prioritize the general principles governing their evolution.

Although the combinatorial interactions are quite complex, the evolution of the SARS-CoV-2 spike protein highlights a relatively straightforward theme: tradeoffs between stability and fusogenicity (72, 73). The combinatorial interactions contribute to the exquisite tuning of spike conformational transitions to suit the environments encountered during infection and transmission. The D614G mutation will receive distinct attention due to its epistatic significance (59). Other mutations, while also important, will be discussed in groups. Most of our discussion will focus on how various mutations influence spike structural dynamics. These changes then have consequences for infectivity and transmission, processes involving viral particles in aggregate. For instance, a variant with a less fusogenic spike can be more transmissible despite having lower titers if it is unlikely to misfire before reaching a new host. Meanwhile, a variant with a more fusogenic spike can be more transmissible if it overcomes the drawbacks of instability through rapid growth kinetics, high titers, and mass action.

Initial studies of the S:D614G spike focused on potentially improved ACE2 affinity (66). However, cryogenic electron microscopy (cryo-EM) experiments revealed that its RBDs were more likely to hold the open conformation, because the S:D614G mutation disrupted an interaction between D614 in the C-terminal domain 2 (CTD2) of the S1 subunit and fusion peptide proximal region (FPPR) of the S2 subunit. Moreover, D614G enabled the first observation of sarbecovirus S trimers with 3 RBDs simultaneously in the open conformation (48). While this finding shed light on how D614G improved ACE2 affinity and made S more susceptible to neutralization, it did not explain why D614G particles were less likely to spontaneously transition to the postfusion conformation (51, 61). Additional cryo-EM studies demonstrated that D614G also caused structural rearrangements near the interface of the Spike protomers. The mutation rigidified the nearby 630 loop of CTD2, allowing it to wedge between the NTD and C-terminal domain 1 (CTD1). This rearrangement of the 630 loop likely strengthened engagement between the S1 and S2 subunits through additional hydrophobic interactions. By slowing overall kinetics, D614G simultaneously slowed firing and increased its tolerance of the 3 RBD open state critical for fusion-enabling S2′ cleavage (49). Thus, the overstabilized D614G spike protein was able to tolerate fusogenicity-enhancing mutations that otherwise would have impaired transmission due to instability-induced misfiring (51).

Arguably the most important mutational hot spot for its multilayered roles in S stability, fusogenicity, transmissibility, and the overall emergence of the COVID-19 pandemic is the S1/S2 furin cleavage site (FCS) (reviewed in reference 74). Because early SARS-CoV-2 isolates were inefficiently processed at S1/S2 (75), an improved FCS would result in viral particles whose spike proteins were more labile and whose RBDs sample the open state more frequently. Although other mutations near the S1/S2 site have been observed (76), the most successful lineages have converged on P681H or P681R (33, 34, 77, 78). Although mutations at the S:677 position may modestly affect furin processing (79), position 681 is the P5 site of the cleavage motif. As furin prefers a basic residue at this site (44), replacement with arginine confers the greatest increase in cleavage (78, 80). However, the transition to the postfusion conformation is irreversible, and multiple simultaneous open RBDs are inherently unstable (48). Thus, S1/S2 sites do not affect fitness in isolation, and there is probably significance to the observation that variants of concern (VOC) differ in their S1/S2 cleavage efficiencies (34).

The hypervariable region of the NTD is another hot spot in which many variants feature deletions and other mutations (81–83). While many studies have addressed their evasion of antibodies given their frequent emergence in immunocompromised patients (83–89), a recent study using SARS-CoV-2 virus-like particles sheds tremendous light on allosteric regulation and epistatic interactions within the spike protein (51). SARS-CoV-1 lacks the S1/S2 furin site of SARS-CoV-2 but possesses deletions in all 3 NTD loops, whereas SARS-CoV-2 variants of concern only possess deletions in 1 or 2 of these loops. By swapping the variable portions of the S1 NTD between SARS-CoV-1, SARS-CoV-2, and SARS-CoV-2 carrying S:D614G, the authors made several key observations. While the spike proteins of SARS-CoV-1 and SARS-CoV-2 showed similar fusogenicity to each other, deletions in the SARS-CoV-2 spike’s NTD loops significantly reduced its fusogenicity. However, the SARS-CoV-2 spike carrying D614G became more fusogenic with NTD loop deletions. Therefore, NTD loop deletions can increase S fusogenicity, but only in the context of the D614G substitution.

The next set of experiments involved centrifuging virus-like particles through sucrose solutions, followed by assessing fusogenicity. Postcentrifugation fusion assays showed that the SARS-CoV-2 D614G spike, which was formerly poorly fusogenic, was now the most fusogenic. However, when NTD deletions were included, the D614G SARS-CoV-2 spike became less fusogenic than the ancestral D614 SARS-CoV-2 spike, i.e., that encoded by hCoV-19/Wuhan/WIV04/2019 (WIV04). Thus, because mutations that increase fusogenicity often decrease stability, a more fusogenic spike protein often results in a less infectious viral particle through irreversible instability-induced misfiring (Fig. 1A).

Given this context, mutations in the RBD are not straightforward. Increased ACE2 binding can be through stronger interactions with the receptor or enhanced RBD opening (49). Similar to D614G and NTD deletions, RBD mutations have important consequences for spike stability. While mutations such as E484K (90–92), N501Y (90), L452R (67), and many others can promote ACE2 binding, they often partially mediate this through local destabilizing effects (91) that allosterically regulate RBD opening via interactions within the S1 subunit. Indeed, a structural remodeling of the RBD that establishes additional salt bridges with ACE2 is one of the distinguishing features of the Omicron variant (93, 94).

Furthermore, there are other convergent mutations within *S* as well as in other viral genes that likely impact fitness but have not yet been thoroughly characterized. For example, multiple SARS-CoV-2 variants carry profusogenic deletions in their cytoplasmic tails (95). Meanwhile, D950N, a signature mutation of the Delta (B.1.617.2 + AY.x) lineage, which is also found in Mu and a number of other variants (35), lies within the HR1 domain (96). Such convergence is a strong indicator of functional relevance and in this case remains largely unexplored. Further, although S protein stability and fusogenicity is an important axis, the *S* gene is not the only part of the genome that affects viral fitness (97). Mutations in the nucleocapsid gene often appear in variants of concern and can contribute to fitness (98, 99), as can those in the putative viroporin encoded by *ORF3A* (100), *ORF8A* (101), and *NSP6* (102, 103), among other genes (104). Moreover, mutations in different genes are capable of epistatic interaction (59). Therefore, each variant exists as a complex and multifaceted configuration.

## ARE HIGHLY OPEN RECEPTOR BINDING DOMAINS AN ADAPTATION TO ZOONOTIC SPILLOVER?

Despite the transmissibility benefits of a stable S protein that tolerates efficient proteolytic processing and predominantly open RBDs, spike proteins of endemic coronaviruses typically favor closed RBDs (105–108). Although SARS-CoV-2 spike variants with more open RBDs showed strong transmission advantages early during the pandemic, the same characteristics that promote receptor binding and rapid entry also increase susceptibility to neutralizing antibodies (48).

Along these lines, the emergence of the S:D614G mutation early during the pandemic set the stage for even more infectious SARS-CoV-2 variants. The D614G S exhibits decreased S1 dissociation after priming (S1/S2 cleavage), which in large part negates the otherwise high fitness costs of frequently sampling the 3 RBD open state necessary for S2′ cleavage. Consequently, the D614G change enabled selection for additional S mutations observed in many variants, with many exhibiting convergent evolution, such as N501Y, E484K, NTD deletions, and P681H/R. Hence, the emergence of D614G provided an epistatic shift in the fitness landscape by increasing the S protein’s tolerance for the 3 RBD open state (51). Although the ancestral Spike encoded by the December 2019 SARS-CoV-2 genome, i.e., WIV04, was sufficiently stable and fusogenic to enable respiratory transmission (32, 72, 109), more efficient proteolytic preprocessing at S1/S2 or a greater propensity toward open RBDs may have been deleterious in this context. In particular, we expect that alteration of proline 681 to either histidine or arginine, which is posited to be critical for the enhanced transmissibility of variant of concern lineages (33–35), would cause instability-induced misfiring in the context of the Dec 2019 WIV04 Spike. In other words, more efficient preprocessing by furin at S1/S2 may confer fitness advantages only when the S2 fusogen is stabilized by mutations found in VOCs (which occur primarily within the S1 subunit).

Moreover, the NTD is an antigenic supersite that is frequently targeted by neutralizing antibodies during primary infection. The structural remodeling induced by NTD loop deletions appears to simultaneously avoid binding of certain classes of NTD-directed antibodies (84, 86, 94) and promote RBD opening (51). Deep sequencing and mathematical modeling suggest that, due to a temporal mismatch between viral replication and antibody production, antigenic evolution can be nearly neutral within the host but highly adaptive at the level of circulating variants (110, 111). Therefore, it seems plausible that a similar process could have played out in the early stages of the SARS-CoV-2 pandemic, in which nearly neutral within-host antigenic evolution to escape NTD and RBD down-directed antibodies facilitated the emergence of variants with NTD loop deletions and a preference for open RBD states. Such variants were even more transmissible at the population level due to rapid growth kinetics in the absence of prior humoral immunity (Fig. 1B).

Overall, the Spike-mediated determinants of coronavirus transmissibility appear to involve a combination of S protein stability, proteolytic processing, propensity for sampling RBD open states necessary to interact with the host receptor (112), the strength of the interaction when such contact occurs, and additional factors, such as the fusion peptide’s capacity for membrane ordering (27, 37). Moreover, we hypothesize that the level of fitness conferred by these features is strongly impacted by the immune status of the host population. S proteins that are efficiently cleaved and frequently sample open RBD states show efficient receptor engagement and S2′ processing. Consequently, variants encoding S proteins with such features spread rapidly within and between seronegative individuals. However, antibodies present in recovered and vaccinated individuals compete with receptors and proteases for these epitopes and can even induce S1 dissociation and misfiring (46). Indeed, it has long been hypothesized that humoral immunity prevents selection of the viral entry protein configurations that would otherwise be the most transmissible in an antibody-deficient population (113).

## CONCLUSIONS

Although the continued emergence of additional SARS-CoV-2 S variants is all but inevitable, it is difficult to predict precisely what changes will occur or to confidently assign time scales. Most changes in S prior to the emergence of the Omicron variant appear to have been driven by selection for improved transmission between immunologically naive hosts. However, this adaptive force should gradually weaken as reservoirs of seronegative individuals are depleted and the virus reaches a state where most transmission is driven by reinfection mediated by antigenic drift and naive infections are limited to young children.

Recent cryo-EM studies of the Omicron S protein suggest that while the Delta Spike predominantly occupies conformations with 1 or more RBDs open simultaneously, the Omicron Spike appears to prefer conformations with 0 or 1 open RBD (94, 114). Additionally, cryo-EM of the Omicron Spike with ACE2 or the S309 antibody occupied only the 1 or 2 open RBD states (93). This contrasts with the D614G Spike, which was also observed in the 3 RBD open state. The increased preference for the closed RBD state in Omicron is partially explained by the introduction of additional electrostatic contacts between the S1 and S2 subunits due to the mutations N764K, T547K, N856K, and N969K. These interactions support a structural basis for the Omicron variant’s increased preference for closed RBDs and decreased S1 shedding (93, 114). Furthermore, these mutations may compensate for the presence of NTD deletions that promote RBD opening and Spike destabilization. Intriguingly, the Omicron variant appears to exhibit less efficient S1/S2 furin cleavage despite the presence of two mutations, P681H and N679K, that individually increase preprocessing at S1/S2 (115, 116). However, a decrease in RBD opening and an increase in overall stability is consistent with impaired S1/S2 cleavage. Further, the L981F mutation appears to improve hydrophobic packing of the S2 subunit, which would likely also contribute to overall stability (93). In contrast, the mutations Q493R, G396S, and Q498R appear to introduce two new salt bridges and one additional hydrogen bond with ACE2 (93, 114). Curiously, the Omicron variant also appears to demonstrate reduced sensitivity to IFITMs (115), which may indicate changes in the function of its fusion peptide, perhaps involving the nearby N856K mutation. Thus, while the Omicron Spike is less likely to occupy the open RBD states necessary for ACE2 engagement, it may compensate by binding the receptor more strongly when such an interaction occurs and by having adapted to resist innate defenses. Such changes would be consistent with the broad pattern of host adaptation suggested by the presence of additional convergent mutations that were observed in prior variants (59, 103).

Further, if the S2′ loop is only accessible for cleavage when the S2 subunit is destabilized, such as in the 3 RBD open state (Fig. 1A), then Omicron’s poor sampling of this conformation would predict a preference for endosomal entry, consistent with its reported phenotype. Moreover, the endosomal pathway may be less reliant on RBD opening and ACE2 binding-mediated destabilization of the S2 subunit. As endosomes mature, they experience an influx of calcium and acidify (117, 118). Low pH does not appear to destabilize coronavirus spike proteins (119) as it does the influenza hemagglutinin (HA) protein (45). However, divalent cations can also destabilize HA (120) and have been long established to influence protein folding and biological membrane curvature by modulating phenomena such as salt bridges, cation-pi interactions, and the hydrophobic effect (121, 122). Lastly, coronavirus fusion peptides require calcium ions to efficiently order membranes (36, 37).

The overall pattern of mutation, structural remodeling, and reduction in binding of antibodies generated against prior Spike structural configurations is consistent with antigenic drift. While most antibodies only recognize RBDs in the up or down state, repeat exposure, either through vaccine booster or breakthrough infection, triggers a memory response and further affinity maturation. Successive rounds of affinity maturation appear to promote the generation and maintenance of broadly neutralizing antibodies, including those capable of recognizing Spike proteins in both the RBD up and RBD down conformations (123, 124).

Moreover, the antigenicity of stabilizing elements such as the SARS-CoV-2 NTD, and the tendency of primary immune responses to generate a relatively limited repertoire of antibodies, may help explain the selection for open RBDs early in the pandemic as well as the subsequent shift in the selective landscape that led to the Omicron variant’s emergence and rapid sweep (125). The Canyon Hypothesis predicts that animal viruses will encode entry proteins that favor closed RBDs at the time of spillover because such viruses have adapted to host humoral immunity, which selects against highly exposed RBDs (113). A particularly intriguing implication when applying this hypothesis to pandemic viruses is that spillover may temporarily free viral entry proteins from a trade-off between infectivity and immune evasion. Moreover, immune responses targeting closed RBD conformations in the early stages of a pandemic may even select for increasingly open entry proteins that enhance transmissibility in seronegative populations (Fig. 1B). Viruses encoding open RBD configurations may spread rapidly due to fast entry kinetics and broad cell tropism but will likely be disfavored over long periods of time due to their inherent instability and susceptibility to neutralization or misfiring.

These principles are also relevant to other preemergent coronaviruses. For example, while MERS has furin recognition motifs at both its S1/S2 and S2′ sites, only S1/S2 is preprocessed. The rapid mode of direct entry at the cell surface is largely restricted to cells that express TMPRSS2 (126). Similarly, introducing a furin motif at the S2′ position of PEDV was unable to mediate S2′ preprocessing or surface entry (127). Artificial overexpression of furin, however, has been observed to enable cell surface entry by MERS in TMPRSS2-deficient cells, albeit inefficiently (128). Given that the MERS S protein has not been observed in the 3 RBD open conformation and has a highly ordered S2′ loop in its 1- and 2-RBD open states (129), its inefficient S2′ preprocessing may be due to infrequent sampling of the fully open state as well as its inherent instability. However, if MERS were to achieve sustained human-human transmission, then improved sampling and tolerance of RBD open states, which the Canyon Hypothesis predicts would be advantageous in the absence of prior humoral immunity, may also improve cleavage at its otherwise cryptic S2′ furin motif. This development would be concerning, as such a configuration may show expanded cell tropism, faster growth, and enhanced cell-to-cell spread. Moreover, acquisition of this feature has been observed during serial *in ovo* passage of avian infectious bronchitis virus, a gammacoronavirus (130).

At least one report demonstrates the expanded tropism possible if the S2′ site is processed before reaching the target cell (131). In this case, a SARS-CoV-2 isolate developed several *S* mutations during serial passage in cultured Vero E6 cells, including a 9-amino-acid NTD deletion as well as E484D, D614G, Q954H, and P812R, the last of which introduces a furin motif at the S2′ site. The authors observed that purified viral particles were competent to initiate rapid growth with extensive syncytia, even in A549 cells, which are deficient for ACE2 and TMPRSS2 (132). This phenotype suggests furin-mediated preprocessing at both the S1/S2 and the S2′ positions, similar to what is seen for the respiratory syncytial virus fusion protein (F) (133, 134), and may further indicate that furin- or TMPRSS2-mediated S2′ cleavage requires frequent RBD opening.

In conclusion, the SARS-CoV-2 Spike protein appears to have followed a general trend of RBD opening and increasing fusogenicity in the early pandemic, with what may now be the beginning of a gradual return to closed RBDs. Population immunity will likely be established as more and more individuals experience vaccination and serial exposures to divergent Spike variants through infection. Iterative rounds of affinity maturation appear to facilitate establishment of expansive antibody repertoires, including broadly neutralizing antibodies capable of recognizing diverse RBDs in both the up (open) and down (closed) conformations (123, 135). These antibodies impose disproportionate fitness costs on open RBDs, leading to an eventual long-term preference for Spike proteins that are stabilized in RBD down (or closed) states (108).

Given the antigenic evolution of seasonal human coronaviruses (136) and the intensity of the Omicron wave, we expect future variants to emerge with reduced sensitivity to the suite of neutralizing antibodies it most commonly elicits. Although Omicron appears to favor the RBD closed state, this does not guarantee that all subsequent variants will follow suit. Fitness landscapes are complex, and evolution is strongly influenced by viral population size (137, 138). Nonetheless, the general trend of RBD opening early in a pandemic, followed by their gradual closing, may help explain the observation that acute respiratory virus pandemics involve escalating waves in the first few years, followed by several years of elevated levels of illness (139–141), which presumably occur in cyclic fashion as population-level immunity is gradually established (Fig. 1B). The complex interactions between viruses, their entry proteins, and the immune systems of the hosts they infect exemplify the stochastic, yet path-dependent, nature of evolution (142–144). These dynamics, coupled with the inherently global nature of pandemics, underscore the importance of swift vaccination in response to emerging viral diseases, which in turn entails equitable and decentralized approaches for viral genomic surveillance and vaccine production. Vaccination, unlike infection-acquired immunity, affords a route to population immunity without viral replication or antigenic evolution (145, 146).
